# Supplementary material for: Community structure of actively growing bacteria in a coastal fish-farming area
Source: PLoS One. 2020 Jun 26;15(6):e0235336. doi: 10.1371/journal.pone.0235336 (PMC7319345; doi:10.1371/journal.pone.0235336)
Supplement: S1 Raw images — (PDF) [file pone.0235336.s002.pdf]

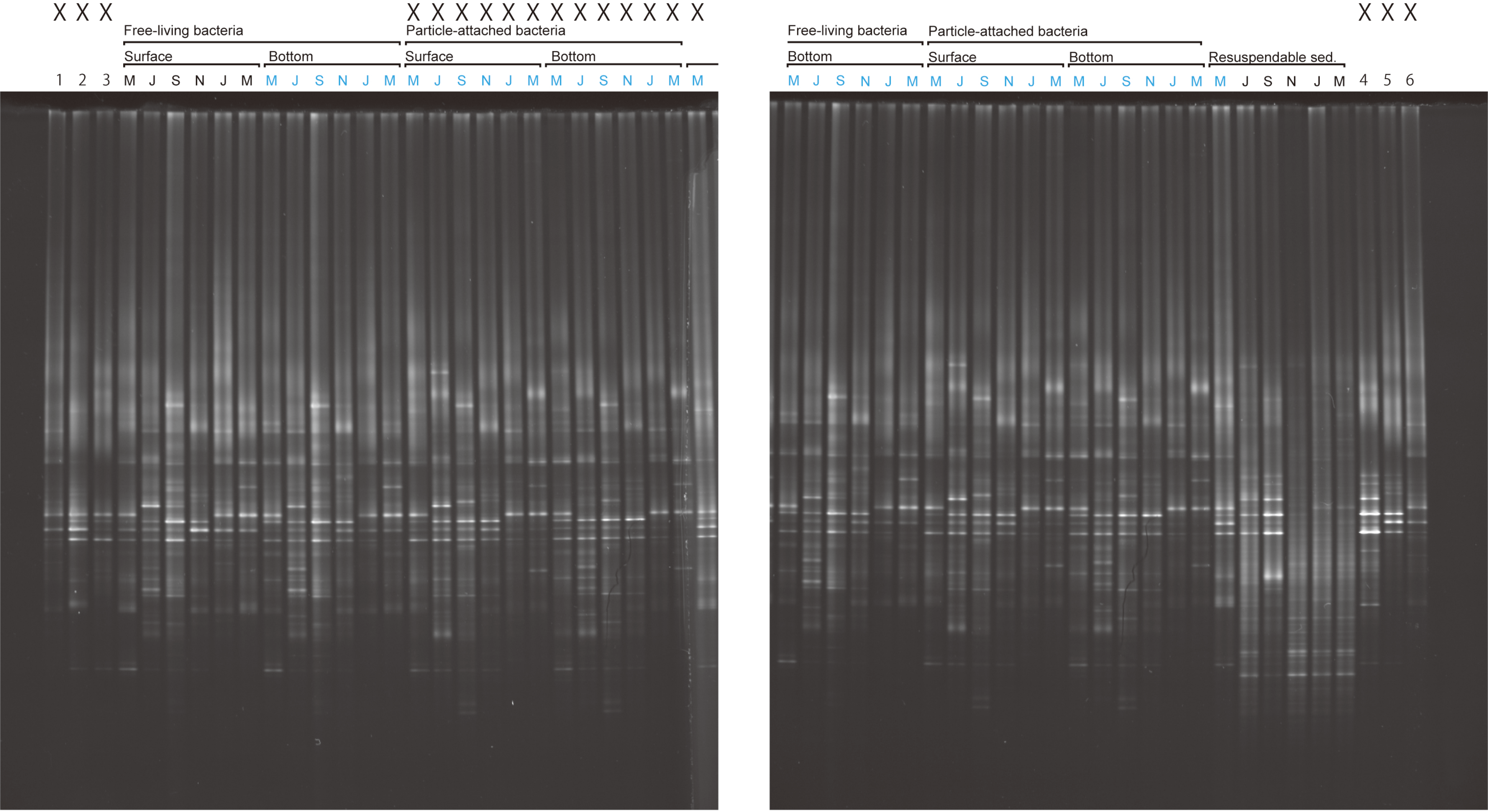

S1 Fig. Raw DGGE gel iamges of 16S rRNA gene amplified from total community in the aquaculture environment.

The gels were visualized and documented using a ChemiDoc imaging system (Bio-Rad). Some of the highlighted lanes in blue overlap because all the panels could not be imaged simultaneously. The lanes with the "X" above the lane label were removed in the final figure (Fig. 2). Samples were collected in May (M), July (J), September (S), November (N), January (J), and March (M), and the corresponding lanes are labeled and ordered accordingly. Lanes 1–6: markers.

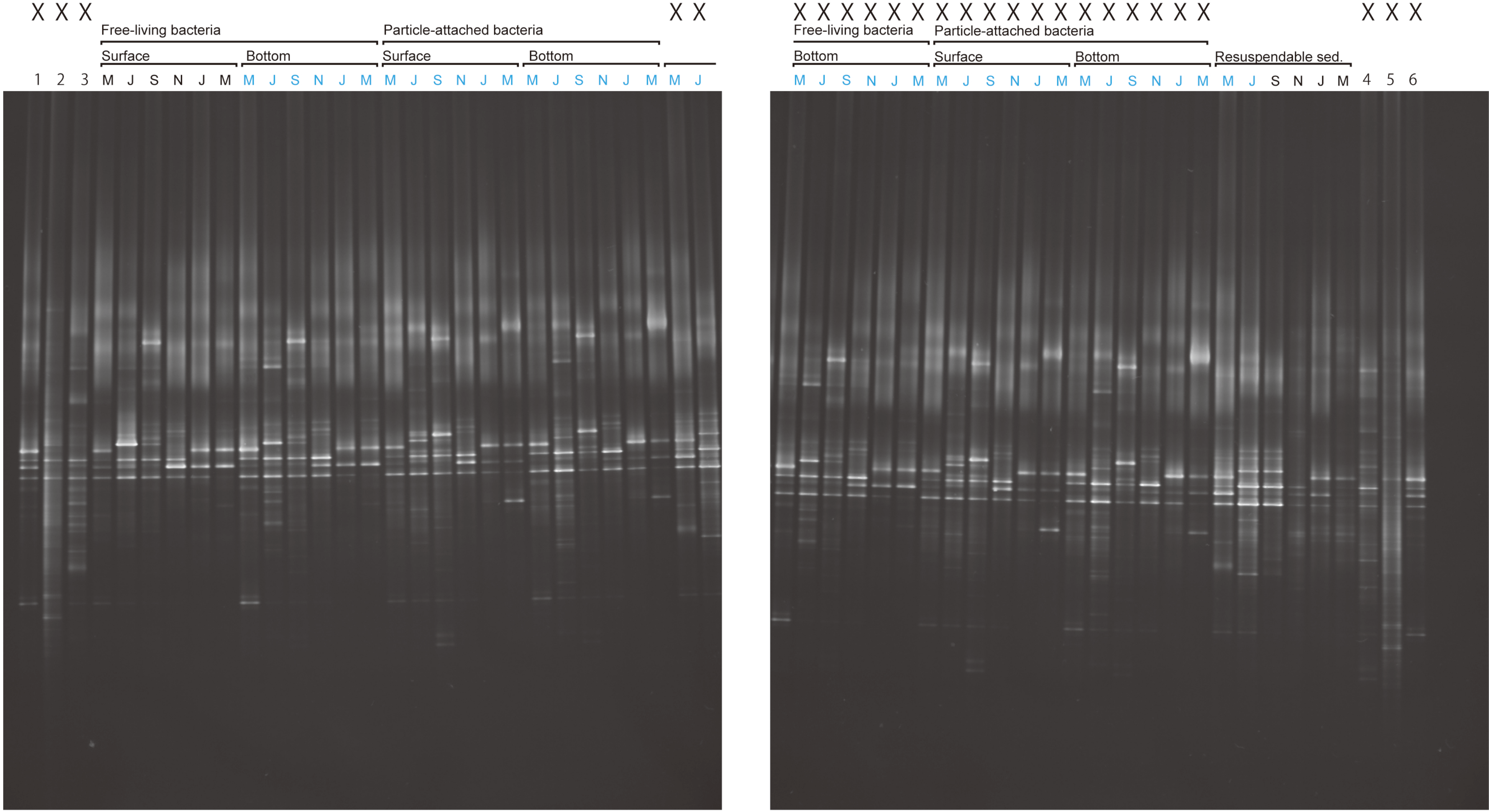

S2 Fig. Raw DGGE gel iamges of 16S rRNA gene amplified from BrdU-incorporating community in the aquaculture environment.

The gels were visualized and documented using a ChemiDoc imaging system (Bio-Rad). Some of the highlighted lanes in blue overlap because all the panels could not be imaged simultaneously. The lanes with the "X" above the lane label were removed in the final figure (Fig. 2). Samples were collected in May (M), July (J), September (S), November (N), January (J), and March (M), and the corresponding lanes are labeled and ordered accordingly. Lanes 1–6: markers.
